# Supplementary figures and images for: Automated system for diagnosing endometrial cancer by adopting deep-learning technology in hysteroscopy
Source: PLoS One. 2021 Mar 31;16(3):e0248526. doi: 10.1371/journal.pone.0248526 (PMC8011803; doi:10.1371/journal.pone.0248526)

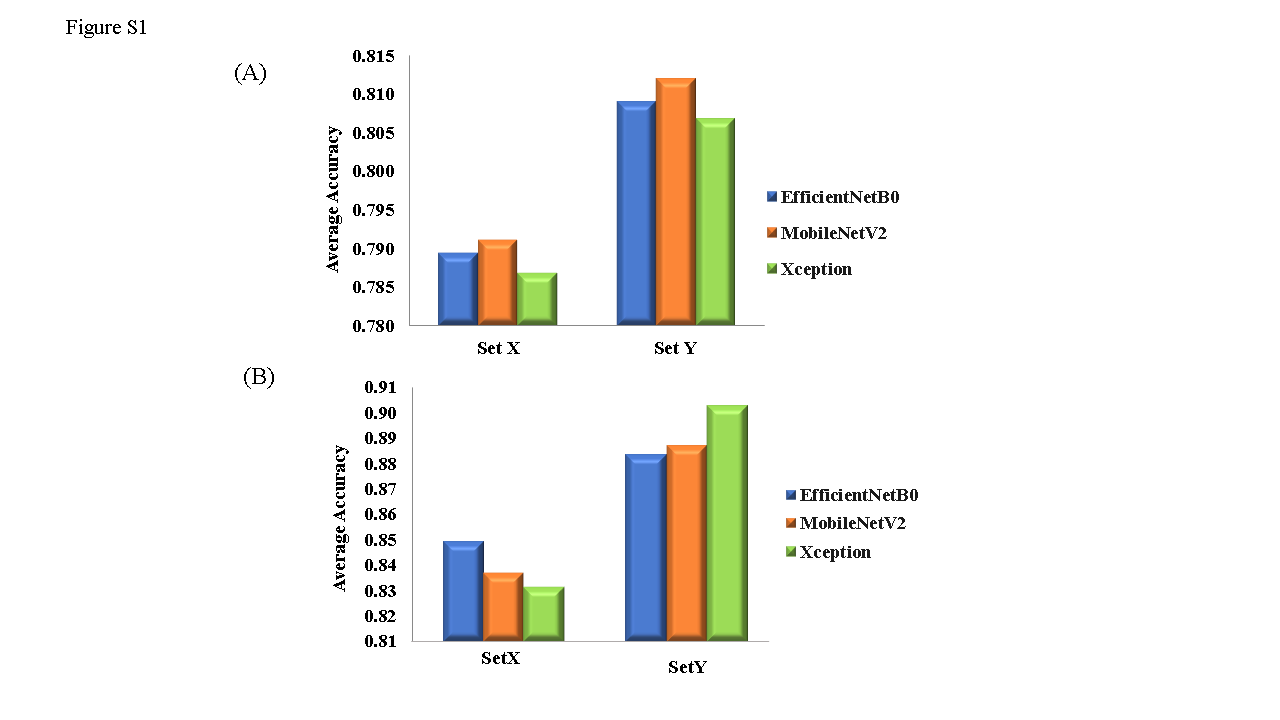

Supplement: S1 Fig — (A) Diagnostic accuracy realized when applying the neural networks on individual datasets. Image-classification accuracy was compared using dataset–neural-network combination. (B) Diagnostic accuracy realized when employing proposed continuity analysis using dataset–neural-network combination. (TIF) [file pone.0248526.s001.TIF]
