# Supplementary material for: Automated system for diagnosing endometrial cancer by adopting deep-learning technology in hysteroscopy
Source: PLoS One. 2021 Mar 31;16(3):e0248526. doi: 10.1371/journal.pone.0248526 (PMC8011803; doi:10.1371/journal.pone.0248526)
Supplement: S1 Table — (DOCX) [file pone.0248526.s002.docx]

**TableS1: Stages and histological types endometrial cancer identified in patients recruited in this study**

|  | TNM | FIGO | Histological type |
| --- | --- | --- | --- |
| 1 | T1bN0M0 | ⅠB | Endometrioid carcinoma G1 |
| 2 | T1aN0M0 | ⅠA | Endometrioid carcinoma G1 |
| 3 | T1aN0M0 | ⅠA | Endometrioid carcinoma G1 |
| 4 | T1aN0M0 | ⅠA | Endometrioid carcinoma G1 |
| 5 | T3bN0M0 | ⅢB | Endometrioid carcinoma G3 |
| 6 | T1aN0M0 | ⅠA | Endometrioid carcinoma G1 |
| 7 | T1aN0M0 | ⅠA | Endometrioid carcinoma G1 |
| 8 | T1aN1M0 | ⅢC1 | Endometrioid carcinoma G1 |
| 9 | T1aN0M0 | ⅠA | Endometrioid carcinoma G1 |
| 10 | T1bN0M0 | ⅠB | Endometrioid carcinoma G1 |
| 11 | T3aN0M0 | ⅢA | Squamous cell carcinoma |
| 12 | T1aN0M0 | ⅠA | Endometrioid carcinoma G1 |
| 13 | T1aN0M0 | ⅠA | Endometrioid carcinoma G3 |
| 14 | T1aN0M0 | ⅠA | Endometrioid carcinoma G1 |
| 15 | T3aN0M0 | ⅢA | Endometrioid carcinoma G1 |
| 16 | T1aN0M0 | ⅠA | Endometrioid carcinoma G1 |
| 17 | T1aN0M0 | ⅠA | Endometrioid carcinoma G2 |
| 18 | T1aN0M0 | ⅠA | Endometrioid carcinoma G1 |
| 19 | T1aN0M0 | ⅠA | Endometrioid carcinoma G1 |
| 20 | T1aN0M0 | ⅠA | Endometrioid carcinoma G1 |
| 21 | T3aN1M0 | ⅢC1 | Serous adenocarcinoma |
