# Supplementary material for: Automated system for diagnosing endometrial cancer by adopting deep-learning technology in hysteroscopy
Source: PLoS One. 2021 Mar 31;16(3):e0248526. doi: 10.1371/journal.pone.0248526 (PMC8011803; doi:10.1371/journal.pone.0248526)
Supplement: S2 Table — (DOCX) [file pone.0248526.s003.docx]

**TableS2: Training and evaluation data in this study.**

|  | Pair-A | | Pair-B | | Pair-C | | Pair-D | |
| --- | --- | --- | --- | --- | --- | --- | --- | --- |
|  | Training | Evaluation | Training | Evaluation | Training | Evaluation | Training | Evaluation |
| AEH | 12 | 3 | 11 | 4 | 11 | 4 | 11 | 4 |
| Cancer | 16 | 5 | 16 | 5 | 16 | 5 | 15 | 6 |
| Myoma | 16 | 5 | 16 | 5 | 16 | 5 | 15 | 6 |
| Polyp | 45 | 15 | 45 | 15 | 45 | 15 | 45 | 15 |
| Normal | 45 | 15 | 45 | 15 | 45 | 15 | 45 | 15 |
