# Supplementary material for: Automated system for diagnosing endometrial cancer by adopting deep-learning technology in hysteroscopy
Source: PLoS One. 2021 Mar 31;16(3):e0248526. doi: 10.1371/journal.pone.0248526 (PMC8011803; doi:10.1371/journal.pone.0248526)
Supplement: S3 Table — (DOCX) [file pone.0248526.s004.docx]

**TableS3 : Datasets used in this study**

| Class | Category | Video numbers | Frame numbers | |
| --- | --- | --- | --- | --- |
|  |  |  | Set X | Set Y |
| Malignant | AEH | 15 | 42146 | 23890 |
|  | Cancer | 21 | 67811 | 32345 |
| Others | Myoma | 21 | 45,037 | |
|  | Polyp | 60 | 143,449 | |
|  | Normal | 60 | 113,357 | |
