# Supplementary material for: Automated system for diagnosing endometrial cancer by adopting deep-learning technology in hysteroscopy
Source: PLoS One. 2021 Mar 31;16(3):e0248526. doi: 10.1371/journal.pone.0248526 (PMC8011803; doi:10.1371/journal.pone.0248526)
Supplement: S4 Table — (DOCX) [file pone.0248526.s005.docx]

**TableS4: Number of parameters of each network**

|  | EfficientNetB0 | MobileNetV2 | Xception |
| --- | --- | --- | --- |
| Trainable params | 5,321,342 | 3,537,666 | 22,907,178 |
| Non-trainable params | 42,016 | 34,112 | 54,528 |
| Total params | 5,363,358 | 3,571,778 | 22,961,706 |
