# Supplementary material for: Automated system for diagnosing endometrial cancer by adopting deep-learning technology in hysteroscopy
Source: PLoS One. 2021 Mar 31;16(3):e0248526. doi: 10.1371/journal.pone.0248526 (PMC8011803; doi:10.1371/journal.pone.0248526)
Supplement: S5 Table — (DOCX) [file pone.0248526.s006.docx]

**TalbeS5: Network Structure of EfficientNet B0**

Model: "EfficientNetB0"

__________________________________________________________________________________________________

Layer (type) Output Shape Param # Connected to

==================================================================================================

input_1 (InputLayer) [(None, 224, 224, 3) 0

__________________________________________________________________________________________________

stem_conv_pad (ZeroPadding2D) (None, 225, 225, 3) 0 input_1[0][0]

__________________________________________________________________________________________________

stem_conv (Conv2D) (None, 112, 112, 32) 864 stem_conv_pad[0][0]

__________________________________________________________________________________________________

stem_bn (BatchNormalization) (None, 112, 112, 32) 128 stem_conv[0][0]

__________________________________________________________________________________________________

stem_activation (Activation) (None, 112, 112, 32) 0 stem_bn[0][0]

__________________________________________________________________________________________________

block1a_dwconv (DepthwiseConv2D (None, 112, 112, 32) 288 stem_activation[0][0]

__________________________________________________________________________________________________

block1a_bn (BatchNormalization) (None, 112, 112, 32) 128 block1a_dwconv[0][0]

__________________________________________________________________________________________________

block1a_activation (Activation) (None, 112, 112, 32) 0 block1a_bn[0][0]

__________________________________________________________________________________________________

block1a_se_squeeze (GlobalAvera (None, 32) 0 block1a_activation[0][0]

__________________________________________________________________________________________________

block1a_se_reshape (Reshape) (None, 1, 1, 32) 0 block1a_se_squeeze[0][0]

__________________________________________________________________________________________________

block1a_se_reduce (Conv2D) (None, 1, 1, 8) 264 block1a_se_reshape[0][0]

__________________________________________________________________________________________________

block1a_se_expand (Conv2D) (None, 1, 1, 32) 288 block1a_se_reduce[0][0]

__________________________________________________________________________________________________

block1a_se_excite (Multiply) (None, 112, 112, 32) 0 block1a_activation[0][0]

block1a_se_expand[0][0]

__________________________________________________________________________________________________

block1a_project_conv (Conv2D) (None, 112, 112, 16) 512 block1a_se_excite[0][0]

__________________________________________________________________________________________________

block1a_project_bn (BatchNormal (None, 112, 112, 16) 64 block1a_project_conv[0][0]

__________________________________________________________________________________________________

block2a_expand_conv (Conv2D) (None, 112, 112, 96) 1536 block1a_project_bn[0][0]

__________________________________________________________________________________________________

block2a_expand_bn (BatchNormali (None, 112, 112, 96) 384 block2a_expand_conv[0][0]

__________________________________________________________________________________________________

block2a_expand_activation (Acti (None, 112, 112, 96) 0 block2a_expand_bn[0][0]

__________________________________________________________________________________________________

block2a_dwconv_pad (ZeroPadding (None, 113, 113, 96) 0 block2a_expand_activation[0][0]

__________________________________________________________________________________________________

block2a_dwconv (DepthwiseConv2D (None, 56, 56, 96) 864 block2a_dwconv_pad[0][0]

__________________________________________________________________________________________________

block2a_bn (BatchNormalization) (None, 56, 56, 96) 384 block2a_dwconv[0][0]

__________________________________________________________________________________________________

block2a_activation (Activation) (None, 56, 56, 96) 0 block2a_bn[0][0]

__________________________________________________________________________________________________

block2a_se_squeeze (GlobalAvera (None, 96) 0 block2a_activation[0][0]

__________________________________________________________________________________________________

block2a_se_reshape (Reshape) (None, 1, 1, 96) 0 block2a_se_squeeze[0][0]

__________________________________________________________________________________________________

block2a_se_reduce (Conv2D) (None, 1, 1, 4) 388 block2a_se_reshape[0][0]

__________________________________________________________________________________________________

block2a_se_expand (Conv2D) (None, 1, 1, 96) 480 block2a_se_reduce[0][0]

__________________________________________________________________________________________________

block2a_se_excite (Multiply) (None, 56, 56, 96) 0 block2a_activation[0][0]

block2a_se_expand[0][0]

__________________________________________________________________________________________________

block2a_project_conv (Conv2D) (None, 56, 56, 24) 2304 block2a_se_excite[0][0]

__________________________________________________________________________________________________

block2a_project_bn (BatchNormal (None, 56, 56, 24) 96 block2a_project_conv[0][0]

__________________________________________________________________________________________________

block2b_expand_conv (Conv2D) (None, 56, 56, 144) 3456 block2a_project_bn[0][0]

__________________________________________________________________________________________________

block2b_expand_bn (BatchNormali (None, 56, 56, 144) 576 block2b_expand_conv[0][0]

__________________________________________________________________________________________________

block2b_expand_activation (Acti (None, 56, 56, 144) 0 block2b_expand_bn[0][0]

__________________________________________________________________________________________________

block2b_dwconv (DepthwiseConv2D (None, 56, 56, 144) 1296 block2b_expand_activation[0][0]

__________________________________________________________________________________________________

block2b_bn (BatchNormalization) (None, 56, 56, 144) 576 block2b_dwconv[0][0]

__________________________________________________________________________________________________

block2b_activation (Activation) (None, 56, 56, 144) 0 block2b_bn[0][0]

__________________________________________________________________________________________________

block2b_se_squeeze (GlobalAvera (None, 144) 0 block2b_activation[0][0]

__________________________________________________________________________________________________

block2b_se_reshape (Reshape) (None, 1, 1, 144) 0 block2b_se_squeeze[0][0]

__________________________________________________________________________________________________

block2b_se_reduce (Conv2D) (None, 1, 1, 6) 870 block2b_se_reshape[0][0]

__________________________________________________________________________________________________

block2b_se_expand (Conv2D) (None, 1, 1, 144) 1008 block2b_se_reduce[0][0]

__________________________________________________________________________________________________

block2b_se_excite (Multiply) (None, 56, 56, 144) 0 block2b_activation[0][0]

block2b_se_expand[0][0]

__________________________________________________________________________________________________

block2b_project_conv (Conv2D) (None, 56, 56, 24) 3456 block2b_se_excite[0][0]

__________________________________________________________________________________________________

block2b_project_bn (BatchNormal (None, 56, 56, 24) 96 block2b_project_conv[0][0]

__________________________________________________________________________________________________

block2b_drop (Dropout) (None, 56, 56, 24) 0 block2b_project_bn[0][0]

__________________________________________________________________________________________________

block2b_add (Add) (None, 56, 56, 24) 0 block2b_drop[0][0]

block2a_project_bn[0][0]

__________________________________________________________________________________________________

block3a_expand_conv (Conv2D) (None, 56, 56, 144) 3456 block2b_add[0][0]

__________________________________________________________________________________________________

block3a_expand_bn (BatchNormali (None, 56, 56, 144) 576 block3a_expand_conv[0][0]

__________________________________________________________________________________________________

block3a_expand_activation (Acti (None, 56, 56, 144) 0 block3a_expand_bn[0][0]

__________________________________________________________________________________________________

block3a_dwconv_pad (ZeroPadding (None, 59, 59, 144) 0 block3a_expand_activation[0][0]

__________________________________________________________________________________________________

block3a_dwconv (DepthwiseConv2D (None, 28, 28, 144) 3600 block3a_dwconv_pad[0][0]

__________________________________________________________________________________________________

block3a_bn (BatchNormalization) (None, 28, 28, 144) 576 block3a_dwconv[0][0]

__________________________________________________________________________________________________

block3a_activation (Activation) (None, 28, 28, 144) 0 block3a_bn[0][0]

__________________________________________________________________________________________________

block3a_se_squeeze (GlobalAvera (None, 144) 0 block3a_activation[0][0]

__________________________________________________________________________________________________

block3a_se_reshape (Reshape) (None, 1, 1, 144) 0 block3a_se_squeeze[0][0]

__________________________________________________________________________________________________

block3a_se_reduce (Conv2D) (None, 1, 1, 6) 870 block3a_se_reshape[0][0]

__________________________________________________________________________________________________

block3a_se_expand (Conv2D) (None, 1, 1, 144) 1008 block3a_se_reduce[0][0]

__________________________________________________________________________________________________

block3a_se_excite (Multiply) (None, 28, 28, 144) 0 block3a_activation[0][0]

block3a_se_expand[0][0]

__________________________________________________________________________________________________

block3a_project_conv (Conv2D) (None, 28, 28, 40) 5760 block3a_se_excite[0][0]

__________________________________________________________________________________________________

block3a_project_bn (BatchNormal (None, 28, 28, 40) 160 block3a_project_conv[0][0]

__________________________________________________________________________________________________

block3b_expand_conv (Conv2D) (None, 28, 28, 240) 9600 block3a_project_bn[0][0]

__________________________________________________________________________________________________

block3b_expand_bn (BatchNormali (None, 28, 28, 240) 960 block3b_expand_conv[0][0]

__________________________________________________________________________________________________

block3b_expand_activation (Acti (None, 28, 28, 240) 0 block3b_expand_bn[0][0]

__________________________________________________________________________________________________

block3b_dwconv (DepthwiseConv2D (None, 28, 28, 240) 6000 block3b_expand_activation[0][0]

__________________________________________________________________________________________________

block3b_bn (BatchNormalization) (None, 28, 28, 240) 960 block3b_dwconv[0][0]

__________________________________________________________________________________________________

block3b_activation (Activation) (None, 28, 28, 240) 0 block3b_bn[0][0]

__________________________________________________________________________________________________

block3b_se_squeeze (GlobalAvera (None, 240) 0 block3b_activation[0][0]

__________________________________________________________________________________________________

block3b_se_reshape (Reshape) (None, 1, 1, 240) 0 block3b_se_squeeze[0][0]

__________________________________________________________________________________________________

block3b_se_reduce (Conv2D) (None, 1, 1, 10) 2410 block3b_se_reshape[0][0]

__________________________________________________________________________________________________

block3b_se_expand (Conv2D) (None, 1, 1, 240) 2640 block3b_se_reduce[0][0]

__________________________________________________________________________________________________

block3b_se_excite (Multiply) (None, 28, 28, 240) 0 block3b_activation[0][0]

block3b_se_expand[0][0]

__________________________________________________________________________________________________

block3b_project_conv (Conv2D) (None, 28, 28, 40) 9600 block3b_se_excite[0][0]

__________________________________________________________________________________________________

block3b_project_bn (BatchNormal (None, 28, 28, 40) 160 block3b_project_conv[0][0]

__________________________________________________________________________________________________

block3b_drop (Dropout) (None, 28, 28, 40) 0 block3b_project_bn[0][0]

__________________________________________________________________________________________________

block3b_add (Add) (None, 28, 28, 40) 0 block3b_drop[0][0]

block3a_project_bn[0][0]

__________________________________________________________________________________________________

block4a_expand_conv (Conv2D) (None, 28, 28, 240) 9600 block3b_add[0][0]

__________________________________________________________________________________________________

block4a_expand_bn (BatchNormali (None, 28, 28, 240) 960 block4a_expand_conv[0][0]

__________________________________________________________________________________________________

block4a_expand_activation (Acti (None, 28, 28, 240) 0 block4a_expand_bn[0][0]

__________________________________________________________________________________________________

block4a_dwconv_pad (ZeroPadding (None, 29, 29, 240) 0 block4a_expand_activation[0][0]

__________________________________________________________________________________________________

block4a_dwconv (DepthwiseConv2D (None, 14, 14, 240) 2160 block4a_dwconv_pad[0][0]

__________________________________________________________________________________________________

block4a_bn (BatchNormalization) (None, 14, 14, 240) 960 block4a_dwconv[0][0]

__________________________________________________________________________________________________

block4a_activation (Activation) (None, 14, 14, 240) 0 block4a_bn[0][0]

__________________________________________________________________________________________________

block4a_se_squeeze (GlobalAvera (None, 240) 0 block4a_activation[0][0]

__________________________________________________________________________________________________

block4a_se_reshape (Reshape) (None, 1, 1, 240) 0 block4a_se_squeeze[0][0]

__________________________________________________________________________________________________

block4a_se_reduce (Conv2D) (None, 1, 1, 10) 2410 block4a_se_reshape[0][0]

__________________________________________________________________________________________________

block4a_se_expand (Conv2D) (None, 1, 1, 240) 2640 block4a_se_reduce[0][0]

__________________________________________________________________________________________________

block4a_se_excite (Multiply) (None, 14, 14, 240) 0 block4a_activation[0][0]

block4a_se_expand[0][0]

__________________________________________________________________________________________________

block4a_project_conv (Conv2D) (None, 14, 14, 80) 19200 block4a_se_excite[0][0]

__________________________________________________________________________________________________

block4a_project_bn (BatchNormal (None, 14, 14, 80) 320 block4a_project_conv[0][0]

__________________________________________________________________________________________________

block4b_expand_conv (Conv2D) (None, 14, 14, 480) 38400 block4a_project_bn[0][0]

__________________________________________________________________________________________________

block4b_expand_bn (BatchNormali (None, 14, 14, 480) 1920 block4b_expand_conv[0][0]

__________________________________________________________________________________________________

block4b_expand_activation (Acti (None, 14, 14, 480) 0 block4b_expand_bn[0][0]

__________________________________________________________________________________________________

block4b_dwconv (DepthwiseConv2D (None, 14, 14, 480) 4320 block4b_expand_activation[0][0]

__________________________________________________________________________________________________

block4b_bn (BatchNormalization) (None, 14, 14, 480) 1920 block4b_dwconv[0][0]

__________________________________________________________________________________________________

block4b_activation (Activation) (None, 14, 14, 480) 0 block4b_bn[0][0]

__________________________________________________________________________________________________

block4b_se_squeeze (GlobalAvera (None, 480) 0 block4b_activation[0][0]

__________________________________________________________________________________________________

block4b_se_reshape (Reshape) (None, 1, 1, 480) 0 block4b_se_squeeze[0][0]

__________________________________________________________________________________________________

block4b_se_reduce (Conv2D) (None, 1, 1, 20) 9620 block4b_se_reshape[0][0]

__________________________________________________________________________________________________

block4b_se_expand (Conv2D) (None, 1, 1, 480) 10080 block4b_se_reduce[0][0]

__________________________________________________________________________________________________

block4b_se_excite (Multiply) (None, 14, 14, 480) 0 block4b_activation[0][0]

block4b_se_expand[0][0]

__________________________________________________________________________________________________

block4b_project_conv (Conv2D) (None, 14, 14, 80) 38400 block4b_se_excite[0][0]

__________________________________________________________________________________________________

block4b_project_bn (BatchNormal (None, 14, 14, 80) 320 block4b_project_conv[0][0]

__________________________________________________________________________________________________

block4b_drop (Dropout) (None, 14, 14, 80) 0 block4b_project_bn[0][0]

__________________________________________________________________________________________________

block4b_add (Add) (None, 14, 14, 80) 0 block4b_drop[0][0]

block4a_project_bn[0][0]

__________________________________________________________________________________________________

block4c_expand_conv (Conv2D) (None, 14, 14, 480) 38400 block4b_add[0][0]

__________________________________________________________________________________________________

block4c_expand_bn (BatchNormali (None, 14, 14, 480) 1920 block4c_expand_conv[0][0]

__________________________________________________________________________________________________

block4c_expand_activation (Acti (None, 14, 14, 480) 0 block4c_expand_bn[0][0]

__________________________________________________________________________________________________

block4c_dwconv (DepthwiseConv2D (None, 14, 14, 480) 4320 block4c_expand_activation[0][0]

__________________________________________________________________________________________________

block4c_bn (BatchNormalization) (None, 14, 14, 480) 1920 block4c_dwconv[0][0]

__________________________________________________________________________________________________

block4c_activation (Activation) (None, 14, 14, 480) 0 block4c_bn[0][0]

__________________________________________________________________________________________________

block4c_se_squeeze (GlobalAvera (None, 480) 0 block4c_activation[0][0]

__________________________________________________________________________________________________

block4c_se_reshape (Reshape) (None, 1, 1, 480) 0 block4c_se_squeeze[0][0]

__________________________________________________________________________________________________

block4c_se_reduce (Conv2D) (None, 1, 1, 20) 9620 block4c_se_reshape[0][0]

__________________________________________________________________________________________________

block4c_se_expand (Conv2D) (None, 1, 1, 480) 10080 block4c_se_reduce[0][0]

__________________________________________________________________________________________________

block4c_se_excite (Multiply) (None, 14, 14, 480) 0 block4c_activation[0][0]

block4c_se_expand[0][0]

__________________________________________________________________________________________________

block4c_project_conv (Conv2D) (None, 14, 14, 80) 38400 block4c_se_excite[0][0]

__________________________________________________________________________________________________

block4c_project_bn (BatchNormal (None, 14, 14, 80) 320 block4c_project_conv[0][0]

__________________________________________________________________________________________________

block4c_drop (Dropout) (None, 14, 14, 80) 0 block4c_project_bn[0][0]

__________________________________________________________________________________________________

block4c_add (Add) (None, 14, 14, 80) 0 block4c_drop[0][0]

block4b_add[0][0]

__________________________________________________________________________________________________

block5a_expand_conv (Conv2D) (None, 14, 14, 480) 38400 block4c_add[0][0]

__________________________________________________________________________________________________

block5a_expand_bn (BatchNormali (None, 14, 14, 480) 1920 block5a_expand_conv[0][0]

__________________________________________________________________________________________________

block5a_expand_activation (Acti (None, 14, 14, 480) 0 block5a_expand_bn[0][0]

__________________________________________________________________________________________________

block5a_dwconv (DepthwiseConv2D (None, 14, 14, 480) 12000 block5a_expand_activation[0][0]

__________________________________________________________________________________________________

block5a_bn (BatchNormalization) (None, 14, 14, 480) 1920 block5a_dwconv[0][0]

__________________________________________________________________________________________________

block5a_activation (Activation) (None, 14, 14, 480) 0 block5a_bn[0][0]

__________________________________________________________________________________________________

block5a_se_squeeze (GlobalAvera (None, 480) 0 block5a_activation[0][0]

__________________________________________________________________________________________________

block5a_se_reshape (Reshape) (None, 1, 1, 480) 0 block5a_se_squeeze[0][0]

__________________________________________________________________________________________________

block5a_se_reduce (Conv2D) (None, 1, 1, 20) 9620 block5a_se_reshape[0][0]

__________________________________________________________________________________________________

block5a_se_expand (Conv2D) (None, 1, 1, 480) 10080 block5a_se_reduce[0][0]

__________________________________________________________________________________________________

block5a_se_excite (Multiply) (None, 14, 14, 480) 0 block5a_activation[0][0]

block5a_se_expand[0][0]

__________________________________________________________________________________________________

block5a_project_conv (Conv2D) (None, 14, 14, 112) 53760 block5a_se_excite[0][0]

__________________________________________________________________________________________________

block5a_project_bn (BatchNormal (None, 14, 14, 112) 448 block5a_project_conv[0][0]

__________________________________________________________________________________________________

block5b_expand_conv (Conv2D) (None, 14, 14, 672) 75264 block5a_project_bn[0][0]

__________________________________________________________________________________________________

block5b_expand_bn (BatchNormali (None, 14, 14, 672) 2688 block5b_expand_conv[0][0]

__________________________________________________________________________________________________

block5b_expand_activation (Acti (None, 14, 14, 672) 0 block5b_expand_bn[0][0]

__________________________________________________________________________________________________

block5b_dwconv (DepthwiseConv2D (None, 14, 14, 672) 16800 block5b_expand_activation[0][0]

__________________________________________________________________________________________________

block5b_bn (BatchNormalization) (None, 14, 14, 672) 2688 block5b_dwconv[0][0]

__________________________________________________________________________________________________

block5b_activation (Activation) (None, 14, 14, 672) 0 block5b_bn[0][0]

__________________________________________________________________________________________________

block5b_se_squeeze (GlobalAvera (None, 672) 0 block5b_activation[0][0]

__________________________________________________________________________________________________

block5b_se_reshape (Reshape) (None, 1, 1, 672) 0 block5b_se_squeeze[0][0]

__________________________________________________________________________________________________

block5b_se_reduce (Conv2D) (None, 1, 1, 28) 18844 block5b_se_reshape[0][0]

__________________________________________________________________________________________________

block5b_se_expand (Conv2D) (None, 1, 1, 672) 19488 block5b_se_reduce[0][0]

__________________________________________________________________________________________________

block5b_se_excite (Multiply) (None, 14, 14, 672) 0 block5b_activation[0][0]

block5b_se_expand[0][0]

__________________________________________________________________________________________________

block5b_project_conv (Conv2D) (None, 14, 14, 112) 75264 block5b_se_excite[0][0]

__________________________________________________________________________________________________

block5b_project_bn (BatchNormal (None, 14, 14, 112) 448 block5b_project_conv[0][0]

__________________________________________________________________________________________________

block5b_drop (Dropout) (None, 14, 14, 112) 0 block5b_project_bn[0][0]

__________________________________________________________________________________________________

block5b_add (Add) (None, 14, 14, 112) 0 block5b_drop[0][0]

block5a_project_bn[0][0]

__________________________________________________________________________________________________

block5c_expand_conv (Conv2D) (None, 14, 14, 672) 75264 block5b_add[0][0]

__________________________________________________________________________________________________

block5c_expand_bn (BatchNormali (None, 14, 14, 672) 2688 block5c_expand_conv[0][0]

__________________________________________________________________________________________________

block5c_expand_activation (Acti (None, 14, 14, 672) 0 block5c_expand_bn[0][0]

__________________________________________________________________________________________________

block5c_dwconv (DepthwiseConv2D (None, 14, 14, 672) 16800 block5c_expand_activation[0][0]

__________________________________________________________________________________________________

block5c_bn (BatchNormalization) (None, 14, 14, 672) 2688 block5c_dwconv[0][0]

__________________________________________________________________________________________________

block5c_activation (Activation) (None, 14, 14, 672) 0 block5c_bn[0][0]

__________________________________________________________________________________________________

block5c_se_squeeze (GlobalAvera (None, 672) 0 block5c_activation[0][0]

__________________________________________________________________________________________________

block5c_se_reshape (Reshape) (None, 1, 1, 672) 0 block5c_se_squeeze[0][0]

__________________________________________________________________________________________________

block5c_se_reduce (Conv2D) (None, 1, 1, 28) 18844 block5c_se_reshape[0][0]

__________________________________________________________________________________________________

block5c_se_expand (Conv2D) (None, 1, 1, 672) 19488 block5c_se_reduce[0][0]

__________________________________________________________________________________________________

block5c_se_excite (Multiply) (None, 14, 14, 672) 0 block5c_activation[0][0]

block5c_se_expand[0][0]

__________________________________________________________________________________________________

block5c_project_conv (Conv2D) (None, 14, 14, 112) 75264 block5c_se_excite[0][0]

__________________________________________________________________________________________________

block5c_project_bn (BatchNormal (None, 14, 14, 112) 448 block5c_project_conv[0][0]

__________________________________________________________________________________________________

block5c_drop (Dropout) (None, 14, 14, 112) 0 block5c_project_bn[0][0]

__________________________________________________________________________________________________

block5c_add (Add) (None, 14, 14, 112) 0 block5c_drop[0][0]

block5b_add[0][0]

__________________________________________________________________________________________________

block6a_expand_conv (Conv2D) (None, 14, 14, 672) 75264 block5c_add[0][0]

__________________________________________________________________________________________________

block6a_expand_bn (BatchNormali (None, 14, 14, 672) 2688 block6a_expand_conv[0][0]

__________________________________________________________________________________________________

block6a_expand_activation (Acti (None, 14, 14, 672) 0 block6a_expand_bn[0][0]

__________________________________________________________________________________________________

block6a_dwconv_pad (ZeroPadding (None, 17, 17, 672) 0 block6a_expand_activation[0][0]

__________________________________________________________________________________________________

block6a_dwconv (DepthwiseConv2D (None, 7, 7, 672) 16800 block6a_dwconv_pad[0][0]

__________________________________________________________________________________________________

block6a_bn (BatchNormalization) (None, 7, 7, 672) 2688 block6a_dwconv[0][0]

__________________________________________________________________________________________________

block6a_activation (Activation) (None, 7, 7, 672) 0 block6a_bn[0][0]

__________________________________________________________________________________________________

block6a_se_squeeze (GlobalAvera (None, 672) 0 block6a_activation[0][0]

__________________________________________________________________________________________________

block6a_se_reshape (Reshape) (None, 1, 1, 672) 0 block6a_se_squeeze[0][0]

__________________________________________________________________________________________________

block6a_se_reduce (Conv2D) (None, 1, 1, 28) 18844 block6a_se_reshape[0][0]

__________________________________________________________________________________________________

block6a_se_expand (Conv2D) (None, 1, 1, 672) 19488 block6a_se_reduce[0][0]

__________________________________________________________________________________________________

block6a_se_excite (Multiply) (None, 7, 7, 672) 0 block6a_activation[0][0]

block6a_se_expand[0][0]

__________________________________________________________________________________________________

block6a_project_conv (Conv2D) (None, 7, 7, 192) 129024 block6a_se_excite[0][0]

__________________________________________________________________________________________________

block6a_project_bn (BatchNormal (None, 7, 7, 192) 768 block6a_project_conv[0][0]

__________________________________________________________________________________________________

block6b_expand_conv (Conv2D) (None, 7, 7, 1152) 221184 block6a_project_bn[0][0]

__________________________________________________________________________________________________

block6b_expand_bn (BatchNormali (None, 7, 7, 1152) 4608 block6b_expand_conv[0][0]

__________________________________________________________________________________________________

block6b_expand_activation (Acti (None, 7, 7, 1152) 0 block6b_expand_bn[0][0]

__________________________________________________________________________________________________

block6b_dwconv (DepthwiseConv2D (None, 7, 7, 1152) 28800 block6b_expand_activation[0][0]

__________________________________________________________________________________________________

block6b_bn (BatchNormalization) (None, 7, 7, 1152) 4608 block6b_dwconv[0][0]

__________________________________________________________________________________________________

block6b_activation (Activation) (None, 7, 7, 1152) 0 block6b_bn[0][0]

__________________________________________________________________________________________________

block6b_se_squeeze (GlobalAvera (None, 1152) 0 block6b_activation[0][0]

__________________________________________________________________________________________________

block6b_se_reshape (Reshape) (None, 1, 1, 1152) 0 block6b_se_squeeze[0][0]

__________________________________________________________________________________________________

block6b_se_reduce (Conv2D) (None, 1, 1, 48) 55344 block6b_se_reshape[0][0]

__________________________________________________________________________________________________

block6b_se_expand (Conv2D) (None, 1, 1, 1152) 56448 block6b_se_reduce[0][0]

__________________________________________________________________________________________________

block6b_se_excite (Multiply) (None, 7, 7, 1152) 0 block6b_activation[0][0]

block6b_se_expand[0][0]

__________________________________________________________________________________________________

block6b_project_conv (Conv2D) (None, 7, 7, 192) 221184 block6b_se_excite[0][0]

__________________________________________________________________________________________________

block6b_project_bn (BatchNormal (None, 7, 7, 192) 768 block6b_project_conv[0][0]

__________________________________________________________________________________________________

block6b_drop (Dropout) (None, 7, 7, 192) 0 block6b_project_bn[0][0]

__________________________________________________________________________________________________

block6b_add (Add) (None, 7, 7, 192) 0 block6b_drop[0][0]

block6a_project_bn[0][0]

__________________________________________________________________________________________________

block6c_expand_conv (Conv2D) (None, 7, 7, 1152) 221184 block6b_add[0][0]

__________________________________________________________________________________________________

block6c_expand_bn (BatchNormali (None, 7, 7, 1152) 4608 block6c_expand_conv[0][0]

__________________________________________________________________________________________________

block6c_expand_activation (Acti (None, 7, 7, 1152) 0 block6c_expand_bn[0][0]

__________________________________________________________________________________________________

block6c_dwconv (DepthwiseConv2D (None, 7, 7, 1152) 28800 block6c_expand_activation[0][0]

__________________________________________________________________________________________________

block6c_bn (BatchNormalization) (None, 7, 7, 1152) 4608 block6c_dwconv[0][0]

__________________________________________________________________________________________________

block6c_activation (Activation) (None, 7, 7, 1152) 0 block6c_bn[0][0]

__________________________________________________________________________________________________

block6c_se_squeeze (GlobalAvera (None, 1152) 0 block6c_activation[0][0]

__________________________________________________________________________________________________

block6c_se_reshape (Reshape) (None, 1, 1, 1152) 0 block6c_se_squeeze[0][0]

__________________________________________________________________________________________________

block6c_se_reduce (Conv2D) (None, 1, 1, 48) 55344 block6c_se_reshape[0][0]

__________________________________________________________________________________________________

block6c_se_expand (Conv2D) (None, 1, 1, 1152) 56448 block6c_se_reduce[0][0]

__________________________________________________________________________________________________

block6c_se_excite (Multiply) (None, 7, 7, 1152) 0 block6c_activation[0][0]

block6c_se_expand[0][0]

__________________________________________________________________________________________________

block6c_project_conv (Conv2D) (None, 7, 7, 192) 221184 block6c_se_excite[0][0]

__________________________________________________________________________________________________

block6c_project_bn (BatchNormal (None, 7, 7, 192) 768 block6c_project_conv[0][0]

__________________________________________________________________________________________________

block6c_drop (Dropout) (None, 7, 7, 192) 0 block6c_project_bn[0][0]

__________________________________________________________________________________________________

block6c_add (Add) (None, 7, 7, 192) 0 block6c_drop[0][0]

block6b_add[0][0]

__________________________________________________________________________________________________

block6d_expand_conv (Conv2D) (None, 7, 7, 1152) 221184 block6c_add[0][0]

__________________________________________________________________________________________________

block6d_expand_bn (BatchNormali (None, 7, 7, 1152) 4608 block6d_expand_conv[0][0]

__________________________________________________________________________________________________

block6d_expand_activation (Acti (None, 7, 7, 1152) 0 block6d_expand_bn[0][0]

__________________________________________________________________________________________________

block6d_dwconv (DepthwiseConv2D (None, 7, 7, 1152) 28800 block6d_expand_activation[0][0]

__________________________________________________________________________________________________

block6d_bn (BatchNormalization) (None, 7, 7, 1152) 4608 block6d_dwconv[0][0]

__________________________________________________________________________________________________

block6d_activation (Activation) (None, 7, 7, 1152) 0 block6d_bn[0][0]

__________________________________________________________________________________________________

block6d_se_squeeze (GlobalAvera (None, 1152) 0 block6d_activation[0][0]

__________________________________________________________________________________________________

block6d_se_reshape (Reshape) (None, 1, 1, 1152) 0 block6d_se_squeeze[0][0]

__________________________________________________________________________________________________

block6d_se_reduce (Conv2D) (None, 1, 1, 48) 55344 block6d_se_reshape[0][0]

__________________________________________________________________________________________________

block6d_se_expand (Conv2D) (None, 1, 1, 1152) 56448 block6d_se_reduce[0][0]

__________________________________________________________________________________________________

block6d_se_excite (Multiply) (None, 7, 7, 1152) 0 block6d_activation[0][0]

block6d_se_expand[0][0]

__________________________________________________________________________________________________

block6d_project_conv (Conv2D) (None, 7, 7, 192) 221184 block6d_se_excite[0][0]

__________________________________________________________________________________________________

block6d_project_bn (BatchNormal (None, 7, 7, 192) 768 block6d_project_conv[0][0]

__________________________________________________________________________________________________

block6d_drop (Dropout) (None, 7, 7, 192) 0 block6d_project_bn[0][0]

__________________________________________________________________________________________________

block6d_add (Add) (None, 7, 7, 192) 0 block6d_drop[0][0]

block6c_add[0][0]

__________________________________________________________________________________________________

block7a_expand_conv (Conv2D) (None, 7, 7, 1152) 221184 block6d_add[0][0]

__________________________________________________________________________________________________

block7a_expand_bn (BatchNormali (None, 7, 7, 1152) 4608 block7a_expand_conv[0][0]

__________________________________________________________________________________________________

block7a_expand_activation (Acti (None, 7, 7, 1152) 0 block7a_expand_bn[0][0]

__________________________________________________________________________________________________

block7a_dwconv (DepthwiseConv2D (None, 7, 7, 1152) 10368 block7a_expand_activation[0][0]

__________________________________________________________________________________________________

block7a_bn (BatchNormalization) (None, 7, 7, 1152) 4608 block7a_dwconv[0][0]

__________________________________________________________________________________________________

block7a_activation (Activation) (None, 7, 7, 1152) 0 block7a_bn[0][0]

__________________________________________________________________________________________________

block7a_se_squeeze (GlobalAvera (None, 1152) 0 block7a_activation[0][0]

__________________________________________________________________________________________________

block7a_se_reshape (Reshape) (None, 1, 1, 1152) 0 block7a_se_squeeze[0][0]

__________________________________________________________________________________________________

block7a_se_reduce (Conv2D) (None, 1, 1, 48) 55344 block7a_se_reshape[0][0]

__________________________________________________________________________________________________

block7a_se_expand (Conv2D) (None, 1, 1, 1152) 56448 block7a_se_reduce[0][0]

__________________________________________________________________________________________________

block7a_se_excite (Multiply) (None, 7, 7, 1152) 0 block7a_activation[0][0]

block7a_se_expand[0][0]

__________________________________________________________________________________________________

block7a_project_conv (Conv2D) (None, 7, 7, 320) 368640 block7a_se_excite[0][0]

__________________________________________________________________________________________________

block7a_project_bn (BatchNormal (None, 7, 7, 320) 1280 block7a_project_conv[0][0]

__________________________________________________________________________________________________

top_conv (Conv2D) (None, 7, 7, 1280) 409600 block7a_project_bn[0][0]

__________________________________________________________________________________________________

top_bn (BatchNormalization) (None, 7, 7, 1280) 5120 top_conv[0][0]

__________________________________________________________________________________________________

top_activation (Activation) (None, 7, 7, 1280) 0 top_bn[0][0]

__________________________________________________________________________________________________

global_average_pooling2d (Globa (None, 1280) 0 top_activation[0][0]

__________________________________________________________________________________________________

dense (Dense) (None, 1024) 1311744 global_average_pooling2d[0][0]

__________________________________________________________________________________________________

dense_1 (Dense) (None, 2) 2050 dense[0][0]

==================================================================================================
