# Supplementary material for: Automated system for diagnosing endometrial cancer by adopting deep-learning technology in hysteroscopy
Source: PLoS One. 2021 Mar 31;16(3):e0248526. doi: 10.1371/journal.pone.0248526 (PMC8011803; doi:10.1371/journal.pone.0248526)
Supplement: S6 Table — (DOCX) [file pone.0248526.s007.docx]

**TableS6：Network Structure of MobileNet V2**

Model: "MobileNetV2"

__________________________________________________________________________________________________

Layer (type) Output Shape Param # Connected to

==================================================================================================

input_1 (InputLayer) [(None, 224, 224, 3) 0

__________________________________________________________________________________________________

Conv1_pad (ZeroPadding2D) (None, 225, 225, 3) 0 input_1[0][0]

__________________________________________________________________________________________________

Conv1 (Conv2D) (None, 112, 112, 32) 864 Conv1_pad[0][0]

__________________________________________________________________________________________________

bn_Conv1 (BatchNormalization) (None, 112, 112, 32) 128 Conv1[0][0]

__________________________________________________________________________________________________

Conv1_relu (ReLU) (None, 112, 112, 32) 0 bn_Conv1[0][0]

__________________________________________________________________________________________________

expanded_conv_depthwise (Depthw (None, 112, 112, 32) 288 Conv1_relu[0][0]

__________________________________________________________________________________________________

expanded_conv_depthwise_BN (Bat (None, 112, 112, 32) 128 expanded_conv_depthwise[0][0]

__________________________________________________________________________________________________

expanded_conv_depthwise_relu (R (None, 112, 112, 32) 0 expanded_conv_depthwise_BN[0][0]

__________________________________________________________________________________________________

expanded_conv_project (Conv2D) (None, 112, 112, 16) 512 expanded_conv_depthwise_relu[0][0

__________________________________________________________________________________________________

expanded_conv_project_BN (Batch (None, 112, 112, 16) 64 expanded_conv_project[0][0]

__________________________________________________________________________________________________

block_1_expand (Conv2D) (None, 112, 112, 96) 1536 expanded_conv_project_BN[0][0]

__________________________________________________________________________________________________

block_1_expand_BN (BatchNormali (None, 112, 112, 96) 384 block_1_expand[0][0]

__________________________________________________________________________________________________

block_1_expand_relu (ReLU) (None, 112, 112, 96) 0 block_1_expand_BN[0][0]

__________________________________________________________________________________________________

block_1_pad (ZeroPadding2D) (None, 113, 113, 96) 0 block_1_expand_relu[0][0]

__________________________________________________________________________________________________

block_1_depthwise (DepthwiseCon (None, 56, 56, 96) 864 block_1_pad[0][0]

__________________________________________________________________________________________________

block_1_depthwise_BN (BatchNorm (None, 56, 56, 96) 384 block_1_depthwise[0][0]

__________________________________________________________________________________________________

block_1_depthwise_relu (ReLU) (None, 56, 56, 96) 0 block_1_depthwise_BN[0][0]

__________________________________________________________________________________________________

block_1_project (Conv2D) (None, 56, 56, 24) 2304 block_1_depthwise_relu[0][0]

**__________________________________________________________________________________________________**

block_1_project_BN (BatchNormal (None, 56, 56, 24) 96 block_1_project[0][0]

**__________________________________________________________________________________________________**

block_2_expand (Conv2D) (None, 56, 56, 144) 3456 block_1_project_BN[0][0]

__________________________________________________________________________________________________

block_2_expand_BN (BatchNormali (None, 56, 56, 144) 576 block_2_expand[0][0]

__________________________________________________________________________________________________

block_2_expand_relu (ReLU) (None, 56, 56, 144) 0 block_2_expand_BN[0][0]

__________________________________________________________________________________________________

block_2_depthwise (DepthwiseCon (None, 56, 56, 144) 1296 block_2_expand_relu[0][0]

__________________________________________________________________________________________________

block_2_depthwise_BN (BatchNorm (None, 56, 56, 144) 576 block_2_depthwise[0][0]

__________________________________________________________________________________________________

block_2_depthwise_relu (ReLU) (None, 56, 56, 144) 0 block_2_depthwise_BN[0][0]

__________________________________________________________________________________________________

block_2_project (Conv2D) (None, 56, 56, 24) 3456 block_2_depthwise_relu[0][0]

__________________________________________________________________________________________________

block_2_project_BN (BatchNormal (None, 56, 56, 24) 96 block_2_project[0][0]

__________________________________________________________________________________________________

block_2_add (Add) (None, 56, 56, 24) 0 block_1_project_BN[0][0]

block_2_project_BN[0][0]

__________________________________________________________________________________________________

block_3_expand (Conv2D) (None, 56, 56, 144) 3456 block_2_add[0][0]

__________________________________________________________________________________________________

block_3_expand_BN (BatchNormali (None, 56, 56, 144) 576 block_3_expand[0][0]

__________________________________________________________________________________________________

block_3_expand_relu (ReLU) (None, 56, 56, 144) 0 block_3_expand_BN[0][0]

__________________________________________________________________________________________________

block_3_pad (ZeroPadding2D) (None, 57, 57, 144) 0 block_3_expand_relu[0][0]

__________________________________________________________________________________________________

block_3_depthwise (DepthwiseCon (None, 28, 28, 144) 1296 block_3_pad[0][0]

__________________________________________________________________________________________________

block_3_depthwise_BN (BatchNorm (None, 28, 28, 144) 576 block_3_depthwise[0][0]

__________________________________________________________________________________________________

block_3_depthwise_relu (ReLU) (None, 28, 28, 144) 0 block_3_depthwise_BN[0][0]

__________________________________________________________________________________________________

block_3_project (Conv2D) (None, 28, 28, 32) 4608 block_3_depthwise_relu[0][0]

__________________________________________________________________________________________________

block_3_project_BN (BatchNormal (None, 28, 28, 32) 128 block_3_project[0][0]

__________________________________________________________________________________________________

block_4_expand (Conv2D) (None, 28, 28, 192) 6144 block_3_project_BN[0][0]

__________________________________________________________________________________________________

block_4_expand_BN (BatchNormali (None, 28, 28, 192) 768 block_4_expand[0][0]

__________________________________________________________________________________________________

block_4_expand_relu (ReLU) (None, 28, 28, 192) 0 block_4_expand_BN[0][0]

__________________________________________________________________________________________________

block_4_depthwise (DepthwiseCon (None, 28, 28, 192) 1728 block_4_expand_relu[0][0]

__________________________________________________________________________________________________

block_4_depthwise_BN (BatchNorm (None, 28, 28, 192) 768 block_4_depthwise[0][0]

__________________________________________________________________________________________________

block_4_depthwise_relu (ReLU) (None, 28, 28, 192) 0 block_4_depthwise_BN[0][0]

__________________________________________________________________________________________________

block_4_project (Conv2D) (None, 28, 28, 32) 6144 block_4_depthwise_relu[0][0]

__________________________________________________________________________________________________

block_4_project_BN (BatchNormal (None, 28, 28, 32) 128 block_4_project[0][0]

__________________________________________________________________________________________________

block_4_add (Add) (None, 28, 28, 32) 0 block_3_project_BN[0][0]

block_4_project_BN[0][0]

__________________________________________________________________________________________________

block_5_expand (Conv2D) (None, 28, 28, 192) 6144 block_4_add[0][0]

__________________________________________________________________________________________________

block_5_expand_BN (BatchNormali (None, 28, 28, 192) 768 block_5_expand[0][0]

__________________________________________________________________________________________________

block_5_expand_relu (ReLU) (None, 28, 28, 192) 0 block_5_expand_BN[0][0]

__________________________________________________________________________________________________

block_5_depthwise (DepthwiseCon (None, 28, 28, 192) 1728 block_5_expand_relu[0][0]

__________________________________________________________________________________________________

block_5_depthwise_BN (BatchNorm (None, 28, 28, 192) 768 block_5_depthwise[0][0]

__________________________________________________________________________________________________

block_5_depthwise_relu (ReLU) (None, 28, 28, 192) 0 block_5_depthwise_BN[0][0]

__________________________________________________________________________________________________

block_5_project (Conv2D) (None, 28, 28, 32) 6144 block_5_depthwise_relu[0][0]

__________________________________________________________________________________________________

block_5_project_BN (BatchNormal (None, 28, 28, 32) 128 block_5_project[0][0]

__________________________________________________________________________________________________

block_5_add (Add) (None, 28, 28, 32) 0 block_4_add[0][0]

block_5_project_BN[0][0]

__________________________________________________________________________________________________

block_6_expand (Conv2D) (None, 28, 28, 192) 6144 block_5_add[0][0]

__________________________________________________________________________________________________

block_6_expand_BN (BatchNormali (None, 28, 28, 192) 768 block_6_expand[0][0]

__________________________________________________________________________________________________

block_6_expand_relu (ReLU) (None, 28, 28, 192) 0 block_6_expand_BN[0][0]

__________________________________________________________________________________________________

block_6_pad (ZeroPadding2D) (None, 29, 29, 192) 0 block_6_expand_relu[0][0]

__________________________________________________________________________________________________

block_6_depthwise (DepthwiseCon (None, 14, 14, 192) 1728 block_6_pad[0][0]

__________________________________________________________________________________________________

block_6_depthwise_BN (BatchNorm (None, 14, 14, 192) 768 block_6_depthwise[0][0]

__________________________________________________________________________________________________

block_6_depthwise_relu (ReLU) (None, 14, 14, 192) 0 block_6_depthwise_BN[0][0]

__________________________________________________________________________________________________

block_6_project (Conv2D) (None, 14, 14, 64) 12288 block_6_depthwise_relu[0][0]

__________________________________________________________________________________________________

block_6_project_BN (BatchNormal (None, 14, 14, 64) 256 block_6_project[0][0]

__________________________________________________________________________________________________

block_7_expand (Conv2D) (None, 14, 14, 384) 24576 block_6_project_BN[0][0]

__________________________________________________________________________________________________

block_7_expand_BN (BatchNormali (None, 14, 14, 384) 1536 block_7_expand[0][0]

__________________________________________________________________________________________________

block_7_expand_relu (ReLU) (None, 14, 14, 384) 0 block_7_expand_BN[0][0]

__________________________________________________________________________________________________

block_7_depthwise (DepthwiseCon (None, 14, 14, 384) 3456 block_7_expand_relu[0][0]

__________________________________________________________________________________________________

block_7_depthwise_BN (BatchNorm (None, 14, 14, 384) 1536 block_7_depthwise[0][0]

__________________________________________________________________________________________________

block_7_depthwise_relu (ReLU) (None, 14, 14, 384) 0 block_7_depthwise_BN[0][0]

__________________________________________________________________________________________________

block_7_project (Conv2D) (None, 14, 14, 64) 24576 block_7_depthwise_relu[0][0]

__________________________________________________________________________________________________

block_7_project_BN (BatchNormal (None, 14, 14, 64) 256 block_7_project[0][0]

__________________________________________________________________________________________________

block_7_add (Add) (None, 14, 14, 64) 0 block_6_project_BN[0][0]

block_7_project_BN[0][0]

__________________________________________________________________________________________________

block_8_expand (Conv2D) (None, 14, 14, 384) 24576 block_7_add[0][0]

__________________________________________________________________________________________________

block_8_expand_BN (BatchNormali (None, 14, 14, 384) 1536 block_8_expand[0][0]

__________________________________________________________________________________________________

block_8_expand_relu (ReLU) (None, 14, 14, 384) 0 block_8_expand_BN[0][0]

__________________________________________________________________________________________________

block_8_depthwise (DepthwiseCon (None, 14, 14, 384) 3456 block_8_expand_relu[0][0]

__________________________________________________________________________________________________

block_8_depthwise_BN (BatchNorm (None, 14, 14, 384) 1536 block_8_depthwise[0][0]

__________________________________________________________________________________________________

block_8_depthwise_relu (ReLU) (None, 14, 14, 384) 0 block_8_depthwise_BN[0][0]

__________________________________________________________________________________________________

block_8_project (Conv2D) (None, 14, 14, 64) 24576 block_8_depthwise_relu[0][0]

__________________________________________________________________________________________________

block_8_project_BN (BatchNormal (None, 14, 14, 64) 256 block_8_project[0][0]

__________________________________________________________________________________________________

block_8_add (Add) (None, 14, 14, 64) 0 block_7_add[0][0]

block_8_project_BN[0][0]

__________________________________________________________________________________________________

block_9_expand (Conv2D) (None, 14, 14, 384) 24576 block_8_add[0][0]

__________________________________________________________________________________________________

block_9_expand_BN (BatchNormali (None, 14, 14, 384) 1536 block_9_expand[0][0]

__________________________________________________________________________________________________

block_9_expand_relu (ReLU) (None, 14, 14, 384) 0 block_9_expand_BN[0][0]

__________________________________________________________________________________________________

block_9_depthwise (DepthwiseCon (None, 14, 14, 384) 3456 block_9_expand_relu[0][0]

__________________________________________________________________________________________________

block_9_depthwise_BN (BatchNorm (None, 14, 14, 384) 1536 block_9_depthwise[0][0]

__________________________________________________________________________________________________

block_9_depthwise_relu (ReLU) (None, 14, 14, 384) 0 block_9_depthwise_BN[0][0]

__________________________________________________________________________________________________

block_9_project (Conv2D) (None, 14, 14, 64) 24576 block_9_depthwise_relu[0][0]

__________________________________________________________________________________________________

block_9_project_BN (BatchNormal (None, 14, 14, 64) 256 block_9_project[0][0]

__________________________________________________________________________________________________

block_9_add (Add) (None, 14, 14, 64) 0 block_8_add[0][0]

block_9_project_BN[0][0]

__________________________________________________________________________________________________

block_10_expand (Conv2D) (None, 14, 14, 384) 24576 block_9_add[0][0]

__________________________________________________________________________________________________

block_10_expand_BN (BatchNormal (None, 14, 14, 384) 1536 block_10_expand[0][0]

__________________________________________________________________________________________________

block_10_expand_relu (ReLU) (None, 14, 14, 384) 0 block_10_expand_BN[0][0]

__________________________________________________________________________________________________

block_10_depthwise (DepthwiseCo (None, 14, 14, 384) 3456 block_10_expand_relu[0][0]

__________________________________________________________________________________________________

block_10_depthwise_BN (BatchNor (None, 14, 14, 384) 1536 block_10_depthwise[0][0]

__________________________________________________________________________________________________

block_10_depthwise_relu (ReLU) (None, 14, 14, 384) 0 block_10_depthwise_BN[0][0]

__________________________________________________________________________________________________

block_10_project (Conv2D) (None, 14, 14, 96) 36864 block_10_depthwise_relu[0][0]

__________________________________________________________________________________________________

block_10_project_BN (BatchNorma (None, 14, 14, 96) 384 block_10_project[0][0]

__________________________________________________________________________________________________

block_11_expand (Conv2D) (None, 14, 14, 576) 55296 block_10_project_BN[0][0]

__________________________________________________________________________________________________

block_11_expand_BN (BatchNormal (None, 14, 14, 576) 2304 block_11_expand[0][0]

__________________________________________________________________________________________________

block_11_expand_relu (ReLU) (None, 14, 14, 576) 0 block_11_expand_BN[0][0]

__________________________________________________________________________________________________

block_11_depthwise (DepthwiseCo (None, 14, 14, 576) 5184 block_11_expand_relu[0][0]

__________________________________________________________________________________________________

block_11_depthwise_BN (BatchNor (None, 14, 14, 576) 2304 block_11_depthwise[0][0]

__________________________________________________________________________________________________

block_11_depthwise_relu (ReLU) (None, 14, 14, 576) 0 block_11_depthwise_BN[0][0]

__________________________________________________________________________________________________

block_11_project (Conv2D) (None, 14, 14, 96) 55296 block_11_depthwise_relu[0][0]

__________________________________________________________________________________________________

block_11_project_BN (BatchNorma (None, 14, 14, 96) 384 block_11_project[0][0]

__________________________________________________________________________________________________

block_11_add (Add) (None, 14, 14, 96) 0 block_10_project_BN[0][0]

block_11_project_BN[0][0]

__________________________________________________________________________________________________

block_12_expand (Conv2D) (None, 14, 14, 576) 55296 block_11_add[0][0]

__________________________________________________________________________________________________

block_12_expand_BN (BatchNormal (None, 14, 14, 576) 2304 block_12_expand[0][0]

__________________________________________________________________________________________________

block_12_expand_relu (ReLU) (None, 14, 14, 576) 0 block_12_expand_BN[0][0]

__________________________________________________________________________________________________

block_12_depthwise (DepthwiseCo (None, 14, 14, 576) 5184 block_12_expand_relu[0][0]

__________________________________________________________________________________________________

block_12_depthwise_BN (BatchNor (None, 14, 14, 576) 2304 block_12_depthwise[0][0]

__________________________________________________________________________________________________

block_12_depthwise_relu (ReLU) (None, 14, 14, 576) 0 block_12_depthwise_BN[0][0]

__________________________________________________________________________________________________

block_12_project (Conv2D) (None, 14, 14, 96) 55296 block_12_depthwise_relu[0][0]

__________________________________________________________________________________________________

block_12_project_BN (BatchNorma (None, 14, 14, 96) 384 block_12_project[0][0]

__________________________________________________________________________________________________

block_12_add (Add) (None, 14, 14, 96) 0 block_11_add[0][0]

block_12_project_BN[0][0]

__________________________________________________________________________________________________

block_13_expand (Conv2D) (None, 14, 14, 576) 55296 block_12_add[0][0]

__________________________________________________________________________________________________

block_13_expand_BN (BatchNormal (None, 14, 14, 576) 2304 block_13_expand[0][0]

__________________________________________________________________________________________________

block_13_expand_relu (ReLU) (None, 14, 14, 576) 0 block_13_expand_BN[0][0]

__________________________________________________________________________________________________

block_13_pad (ZeroPadding2D) (None, 15, 15, 576) 0 block_13_expand_relu[0][0]

__________________________________________________________________________________________________

block_13_depthwise (DepthwiseCo (None, 7, 7, 576) 5184 block_13_pad[0][0]

__________________________________________________________________________________________________

block_13_depthwise_BN (BatchNor (None, 7, 7, 576) 2304 block_13_depthwise[0][0]

__________________________________________________________________________________________________

block_13_depthwise_relu (ReLU) (None, 7, 7, 576) 0 block_13_depthwise_BN[0][0]

__________________________________________________________________________________________________

block_13_project (Conv2D) (None, 7, 7, 160) 92160 block_13_depthwise_relu[0][0]

__________________________________________________________________________________________________

block_13_project_BN (BatchNorma (None, 7, 7, 160) 640 block_13_project[0][0]

__________________________________________________________________________________________________

block_14_expand (Conv2D) (None, 7, 7, 960) 153600 block_13_project_BN[0][0]

__________________________________________________________________________________________________

block_14_expand_BN (BatchNormal (None, 7, 7, 960) 3840 block_14_expand[0][0]

__________________________________________________________________________________________________

block_14_expand_relu (ReLU) (None, 7, 7, 960) 0 block_14_expand_BN[0][0]

__________________________________________________________________________________________________

block_14_depthwise (DepthwiseCo (None, 7, 7, 960) 8640 block_14_expand_relu[0][0]

__________________________________________________________________________________________________

block_14_depthwise_BN (BatchNor (None, 7, 7, 960) 3840 block_14_depthwise[0][0]

__________________________________________________________________________________________________

block_14_depthwise_relu (ReLU) (None, 7, 7, 960) 0 block_14_depthwise_BN[0][0]

__________________________________________________________________________________________________

block_14_project (Conv2D) (None, 7, 7, 160) 153600 block_14_depthwise_relu[0][0]

__________________________________________________________________________________________________

block_14_project_BN (BatchNorma (None, 7, 7, 160) 640 block_14_project[0][0]

__________________________________________________________________________________________________

block_14_add (Add) (None, 7, 7, 160) 0 block_13_project_BN[0][0]

block_14_project_BN[0][0]

__________________________________________________________________________________________________

block_15_expand (Conv2D) (None, 7, 7, 960) 153600 block_14_add[0][0]

__________________________________________________________________________________________________

block_15_expand_BN (BatchNormal (None, 7, 7, 960) 3840 block_15_expand[0][0]

__________________________________________________________________________________________________

block_15_expand_relu (ReLU) (None, 7, 7, 960) 0 block_15_expand_BN[0][0]

__________________________________________________________________________________________________

block_15_depthwise (DepthwiseCo (None, 7, 7, 960) 8640 block_15_expand_relu[0][0]

__________________________________________________________________________________________________

block_15_depthwise_BN (BatchNor (None, 7, 7, 960) 3840 block_15_depthwise[0][0]

__________________________________________________________________________________________________

block_15_depthwise_relu (ReLU) (None, 7, 7, 960) 0 block_15_depthwise_BN[0][0]

__________________________________________________________________________________________________

block_15_project (Conv2D) (None, 7, 7, 160) 153600 block_15_depthwise_relu[0][0]

__________________________________________________________________________________________________

block_15_project_BN (BatchNorma (None, 7, 7, 160) 640 block_15_project[0][0]

__________________________________________________________________________________________________

block_15_add (Add) (None, 7, 7, 160) 0 block_14_add[0][0]

block_15_project_BN[0][0]

__________________________________________________________________________________________________

block_16_expand (Conv2D) (None, 7, 7, 960) 153600 block_15_add[0][0]

__________________________________________________________________________________________________

block_16_expand_BN (BatchNormal (None, 7, 7, 960) 3840 block_16_expand[0][0]

__________________________________________________________________________________________________

block_16_expand_relu (ReLU) (None, 7, 7, 960) 0 block_16_expand_BN[0][0]

__________________________________________________________________________________________________

block_16_depthwise (DepthwiseCo (None, 7, 7, 960) 8640 block_16_expand_relu[0][0]

__________________________________________________________________________________________________

block_16_depthwise_BN (BatchNor (None, 7, 7, 960) 3840 block_16_depthwise[0][0]

__________________________________________________________________________________________________

block_16_depthwise_relu (ReLU) (None, 7, 7, 960) 0 block_16_depthwise_BN[0][0]

__________________________________________________________________________________________________

block_16_project (Conv2D) (None, 7, 7, 320) 307200 block_16_depthwise_relu[0][0]

__________________________________________________________________________________________________

block_16_project_BN (BatchNorma (None, 7, 7, 320) 1280 block_16_project[0][0]

__________________________________________________________________________________________________

Conv_1 (Conv2D) (None, 7, 7, 1280) 409600 block_16_project_BN[0][0]

__________________________________________________________________________________________________

Conv_1_bn (BatchNormalization) (None, 7, 7, 1280) 5120 Conv_1[0][0]

__________________________________________________________________________________________________

out_relu (ReLU) (None, 7, 7, 1280) 0 Conv_1_bn[0][0]

__________________________________________________________________________________________________

global_average_pooling2d (Globa (None, 1280) 0 out_relu[0][0]

__________________________________________________________________________________________________

dense (Dense) (None, 1024) 1311744 global_average_pooling2d[0][0]

__________________________________________________________________________________________________

dense_1 (Dense) (None, 2) 2050 dense[0][0]

==================================================================================================
