# Supplementary material for: Automated system for diagnosing endometrial cancer by adopting deep-learning technology in hysteroscopy
Source: PLoS One. 2021 Mar 31;16(3):e0248526. doi: 10.1371/journal.pone.0248526 (PMC8011803; doi:10.1371/journal.pone.0248526)
Supplement: S7 Table — (DOCX) [file pone.0248526.s008.docx]

**TableS7：Network Structure of Xception**

Model: "Xception"

__________________________________________________________________________________________________

Layer (type) Output Shape Param # Connected to

==================================================================================================

input_1 (InputLayer) [(None, 256, 256, 3) 0

__________________________________________________________________________________________________

block1_conv1 (Conv2D) (None, 127, 127, 32) 864 input_1[0][0]

__________________________________________________________________________________________________

block1_conv1_bn (BatchNormaliza (None, 127, 127, 32) 128 block1_conv1[0][0]

__________________________________________________________________________________________________

block1_conv1_act (Activation) (None, 127, 127, 32) 0 block1_conv1_bn[0][0]

__________________________________________________________________________________________________

block1_conv2 (Conv2D) (None, 125, 125, 64) 18432 block1_conv1_act[0][0]

__________________________________________________________________________________________________

block1_conv2_bn (BatchNormaliza (None, 125, 125, 64) 256 block1_conv2[0][0]

__________________________________________________________________________________________________

block1_conv2_act (Activation) (None, 125, 125, 64) 0 block1_conv2_bn[0][0]

__________________________________________________________________________________________________

block2_sepconv1 (SeparableConv2 (None, 125, 125, 128 8768 block1_conv2_act[0][0]

__________________________________________________________________________________________________

block2_sepconv1_bn (BatchNormal (None, 125, 125, 128 512 block2_sepconv1[0][0]

__________________________________________________________________________________________________

block2_sepconv2_act (Activation (None, 125, 125, 128 0 block2_sepconv1_bn[0][0]

__________________________________________________________________________________________________

block2_sepconv2 (SeparableConv2 (None, 125, 125, 128 17536 block2_sepconv2_act[0][0]

__________________________________________________________________________________________________

block2_sepconv2_bn (BatchNormal (None, 125, 125, 128 512 block2_sepconv2[0][0]

__________________________________________________________________________________________________

conv2d (Conv2D) (None, 63, 63, 128) 8192 block1_conv2_act[0][0]

__________________________________________________________________________________________________

block2_pool (MaxPooling2D) (None, 63, 63, 128) 0 block2_sepconv2_bn[0][0]

__________________________________________________________________________________________________

batch_normalization (BatchNorma (None, 63, 63, 128) 512 conv2d[0][0]

__________________________________________________________________________________________________

add (Add) (None, 63, 63, 128) 0 block2_pool[0][0]

batch_normalization[0][0]

__________________________________________________________________________________________________

block3_sepconv1_act (Activation (None, 63, 63, 128) 0 add[0][0]

__________________________________________________________________________________________________

block3_sepconv1 (SeparableConv2 (None, 63, 63, 256) 33920 block3_sepconv1_act[0][0]

__________________________________________________________________________________________________

block3_sepconv1_bn (BatchNormal (None, 63, 63, 256) 1024 block3_sepconv1[0][0]

__________________________________________________________________________________________________

block3_sepconv2_act (Activation (None, 63, 63, 256) 0 block3_sepconv1_bn[0][0]

__________________________________________________________________________________________________

block3_sepconv2 (SeparableConv2 (None, 63, 63, 256) 67840 block3_sepconv2_act[0][0]

__________________________________________________________________________________________________

block3_sepconv2_bn (BatchNormal (None, 63, 63, 256) 1024 block3_sepconv2[0][0]

__________________________________________________________________________________________________

conv2d_1 (Conv2D) (None, 32, 32, 256) 32768 add[0][0]

__________________________________________________________________________________________________

block3_pool (MaxPooling2D) (None, 32, 32, 256) 0 block3_sepconv2_bn[0][0]

__________________________________________________________________________________________________

batch_normalization_1 (BatchNor (None, 32, 32, 256) 1024 conv2d_1[0][0]

__________________________________________________________________________________________________

add_1 (Add) (None, 32, 32, 256) 0 block3_pool[0][0]

batch_normalization_1[0][0]

__________________________________________________________________________________________________

block4_sepconv1_act (Activation (None, 32, 32, 256) 0 add_1[0][0]

__________________________________________________________________________________________________

block4_sepconv1 (SeparableConv2 (None, 32, 32, 728) 188672 block4_sepconv1_act[0][0]

__________________________________________________________________________________________________

block4_sepconv1_bn (BatchNormal (None, 32, 32, 728) 2912 block4_sepconv1[0][0]

__________________________________________________________________________________________________

block4_sepconv2_act (Activation (None, 32, 32, 728) 0 block4_sepconv1_bn[0][0]

__________________________________________________________________________________________________

block4_sepconv2 (SeparableConv2 (None, 32, 32, 728) 536536 block4_sepconv2_act[0][0]

__________________________________________________________________________________________________

block4_sepconv2_bn (BatchNormal (None, 32, 32, 728) 2912 block4_sepconv2[0][0]

__________________________________________________________________________________________________

conv2d_2 (Conv2D) (None, 16, 16, 728) 186368 add_1[0][0]

__________________________________________________________________________________________________

block4_pool (MaxPooling2D) (None, 16, 16, 728) 0 block4_sepconv2_bn[0][0]

__________________________________________________________________________________________________

batch_normalization_2 (BatchNor (None, 16, 16, 728) 2912 conv2d_2[0][0]

__________________________________________________________________________________________________

add_2 (Add) (None, 16, 16, 728) 0 block4_pool[0][0]

batch_normalization_2[0][0]

__________________________________________________________________________________________________

block5_sepconv1_act (Activation (None, 16, 16, 728) 0 add_2[0][0]

__________________________________________________________________________________________________

block5_sepconv1 (SeparableConv2 (None, 16, 16, 728) 536536 block5_sepconv1_act[0][0]

__________________________________________________________________________________________________

block5_sepconv1_bn (BatchNormal (None, 16, 16, 728) 2912 block5_sepconv1[0][0]

__________________________________________________________________________________________________

block5_sepconv2_act (Activation (None, 16, 16, 728) 0 block5_sepconv1_bn[0][0]

__________________________________________________________________________________________________

block5_sepconv2 (SeparableConv2 (None, 16, 16, 728) 536536 block5_sepconv2_act[0][0]

__________________________________________________________________________________________________

block5_sepconv2_bn (BatchNormal (None, 16, 16, 728) 2912 block5_sepconv2[0][0]

__________________________________________________________________________________________________

block5_sepconv3_act (Activation (None, 16, 16, 728) 0 block5_sepconv2_bn[0][0]

__________________________________________________________________________________________________

block5_sepconv3 (SeparableConv2 (None, 16, 16, 728) 536536 block5_sepconv3_act[0][0]

__________________________________________________________________________________________________

block5_sepconv3_bn (BatchNormal (None, 16, 16, 728) 2912 block5_sepconv3[0][0]

__________________________________________________________________________________________________

add_3 (Add) (None, 16, 16, 728) 0 block5_sepconv3_bn[0][0]

add_2[0][0]

__________________________________________________________________________________________________

block6_sepconv1_act (Activation (None, 16, 16, 728) 0 add_3[0][0]

__________________________________________________________________________________________________

block6_sepconv1 (SeparableConv2 (None, 16, 16, 728) 536536 block6_sepconv1_act[0][0]

__________________________________________________________________________________________________

block6_sepconv1_bn (BatchNormal (None, 16, 16, 728) 2912 block6_sepconv1[0][0]

__________________________________________________________________________________________________

block6_sepconv2_act (Activation (None, 16, 16, 728) 0 block6_sepconv1_bn[0][0]

__________________________________________________________________________________________________

block6_sepconv2 (SeparableConv2 (None, 16, 16, 728) 536536 block6_sepconv2_act[0][0]

__________________________________________________________________________________________________

block6_sepconv2_bn (BatchNormal (None, 16, 16, 728) 2912 block6_sepconv2[0][0]

__________________________________________________________________________________________________

block6_sepconv3_act (Activation (None, 16, 16, 728) 0 block6_sepconv2_bn[0][0]

__________________________________________________________________________________________________

block6_sepconv3 (SeparableConv2 (None, 16, 16, 728) 536536 block6_sepconv3_act[0][0]

__________________________________________________________________________________________________

block6_sepconv3_bn (BatchNormal (None, 16, 16, 728) 2912 block6_sepconv3[0][0]

__________________________________________________________________________________________________

add_4 (Add) (None, 16, 16, 728) 0 block6_sepconv3_bn[0][0]

add_3[0][0]

__________________________________________________________________________________________________

block7_sepconv1_act (Activation (None, 16, 16, 728) 0 add_4[0][0]

__________________________________________________________________________________________________

block7_sepconv1 (SeparableConv2 (None, 16, 16, 728) 536536 block7_sepconv1_act[0][0]

__________________________________________________________________________________________________

block7_sepconv1_bn (BatchNormal (None, 16, 16, 728) 2912 block7_sepconv1[0][0]

__________________________________________________________________________________________________

block7_sepconv2_act (Activation (None, 16, 16, 728) 0 block7_sepconv1_bn[0][0]

__________________________________________________________________________________________________

block7_sepconv2 (SeparableConv2 (None, 16, 16, 728) 536536 block7_sepconv2_act[0][0]

__________________________________________________________________________________________________

block7_sepconv2_bn (BatchNormal (None, 16, 16, 728) 2912 block7_sepconv2[0][0]

__________________________________________________________________________________________________

block7_sepconv3_act (Activation (None, 16, 16, 728) 0 block7_sepconv2_bn[0][0]

__________________________________________________________________________________________________

block7_sepconv3 (SeparableConv2 (None, 16, 16, 728) 536536 block7_sepconv3_act[0][0]

__________________________________________________________________________________________________

block7_sepconv3_bn (BatchNormal (None, 16, 16, 728) 2912 block7_sepconv3[0][0]

__________________________________________________________________________________________________

add_5 (Add) (None, 16, 16, 728) 0 block7_sepconv3_bn[0][0]

add_4[0][0]

__________________________________________________________________________________________________

block8_sepconv1_act (Activation (None, 16, 16, 728) 0 add_5[0][0]

__________________________________________________________________________________________________

block8_sepconv1 (SeparableConv2 (None, 16, 16, 728) 536536 block8_sepconv1_act[0][0]

__________________________________________________________________________________________________

block8_sepconv1_bn (BatchNormal (None, 16, 16, 728) 2912 block8_sepconv1[0][0]

__________________________________________________________________________________________________

block8_sepconv2_act (Activation (None, 16, 16, 728) 0 block8_sepconv1_bn[0][0]

__________________________________________________________________________________________________

block8_sepconv2 (SeparableConv2 (None, 16, 16, 728) 536536 block8_sepconv2_act[0][0]

__________________________________________________________________________________________________

block8_sepconv2_bn (BatchNormal (None, 16, 16, 728) 2912 block8_sepconv2[0][0]

__________________________________________________________________________________________________

block8_sepconv3_act (Activation (None, 16, 16, 728) 0 block8_sepconv2_bn[0][0]

__________________________________________________________________________________________________

block8_sepconv3 (SeparableConv2 (None, 16, 16, 728) 536536 block8_sepconv3_act[0][0]

__________________________________________________________________________________________________

block8_sepconv3_bn (BatchNormal (None, 16, 16, 728) 2912 block8_sepconv3[0][0]

__________________________________________________________________________________________________

add_6 (Add) (None, 16, 16, 728) 0 block8_sepconv3_bn[0][0]

add_5[0][0]

__________________________________________________________________________________________________

block9_sepconv1_act (Activation (None, 16, 16, 728) 0 add_6[0][0]

__________________________________________________________________________________________________

block9_sepconv1 (SeparableConv2 (None, 16, 16, 728) 536536 block9_sepconv1_act[0][0]

__________________________________________________________________________________________________

block9_sepconv1_bn (BatchNormal (None, 16, 16, 728) 2912 block9_sepconv1[0][0]

__________________________________________________________________________________________________

block9_sepconv2_act (Activation (None, 16, 16, 728) 0 block9_sepconv1_bn[0][0]

__________________________________________________________________________________________________

block9_sepconv2 (SeparableConv2 (None, 16, 16, 728) 536536 block9_sepconv2_act[0][0]

__________________________________________________________________________________________________

block9_sepconv2_bn (BatchNormal (None, 16, 16, 728) 2912 block9_sepconv2[0][0]

__________________________________________________________________________________________________

block9_sepconv3_act (Activation (None, 16, 16, 728) 0 block9_sepconv2_bn[0][0]

__________________________________________________________________________________________________

block9_sepconv3 (SeparableConv2 (None, 16, 16, 728) 536536 block9_sepconv3_act[0][0]

__________________________________________________________________________________________________

block9_sepconv3_bn (BatchNormal (None, 16, 16, 728) 2912 block9_sepconv3[0][0]

__________________________________________________________________________________________________

add_7 (Add) (None, 16, 16, 728) 0 block9_sepconv3_bn[0][0]

add_6[0][0]

__________________________________________________________________________________________________

block10_sepconv1_act (Activatio (None, 16, 16, 728) 0 add_7[0][0]

__________________________________________________________________________________________________

block10_sepconv1 (SeparableConv (None, 16, 16, 728) 536536 block10_sepconv1_act[0][0]

__________________________________________________________________________________________________

block10_sepconv1_bn (BatchNorma (None, 16, 16, 728) 2912 block10_sepconv1[0][0]

__________________________________________________________________________________________________

block10_sepconv2_act (Activatio (None, 16, 16, 728) 0 block10_sepconv1_bn[0][0]

__________________________________________________________________________________________________

block10_sepconv2 (SeparableConv (None, 16, 16, 728) 536536 block10_sepconv2_act[0][0]

__________________________________________________________________________________________________

block10_sepconv2_bn (BatchNorma (None, 16, 16, 728) 2912 block10_sepconv2[0][0]

__________________________________________________________________________________________________

block10_sepconv3_act (Activatio (None, 16, 16, 728) 0 block10_sepconv2_bn[0][0]

__________________________________________________________________________________________________

block10_sepconv3 (SeparableConv (None, 16, 16, 728) 536536 block10_sepconv3_act[0][0]

__________________________________________________________________________________________________

block10_sepconv3_bn (BatchNorma (None, 16, 16, 728) 2912 block10_sepconv3[0][0]

__________________________________________________________________________________________________

add_8 (Add) (None, 16, 16, 728) 0 block10_sepconv3_bn[0][0]

add_7[0][0]

__________________________________________________________________________________________________

block11_sepconv1_act (Activatio (None, 16, 16, 728) 0 add_8[0][0]

__________________________________________________________________________________________________

block11_sepconv1 (SeparableConv (None, 16, 16, 728) 536536 block11_sepconv1_act[0][0]

__________________________________________________________________________________________________

block11_sepconv1_bn (BatchNorma (None, 16, 16, 728) 2912 block11_sepconv1[0][0]

__________________________________________________________________________________________________

block11_sepconv2_act (Activatio (None, 16, 16, 728) 0 block11_sepconv1_bn[0][0]

__________________________________________________________________________________________________

block11_sepconv2 (SeparableConv (None, 16, 16, 728) 536536 block11_sepconv2_act[0][0]

__________________________________________________________________________________________________

block11_sepconv2_bn (BatchNorma (None, 16, 16, 728) 2912 block11_sepconv2[0][0]

__________________________________________________________________________________________________

block11_sepconv3_act (Activatio (None, 16, 16, 728) 0 block11_sepconv2_bn[0][0]

__________________________________________________________________________________________________

block11_sepconv3 (SeparableConv (None, 16, 16, 728) 536536 block11_sepconv3_act[0][0]

__________________________________________________________________________________________________

block11_sepconv3_bn (BatchNorma (None, 16, 16, 728) 2912 block11_sepconv3[0][0]

__________________________________________________________________________________________________

add_9 (Add) (None, 16, 16, 728) 0 block11_sepconv3_bn[0][0]

add_8[0][0]

__________________________________________________________________________________________________

block12_sepconv1_act (Activatio (None, 16, 16, 728) 0 add_9[0][0]

__________________________________________________________________________________________________

block12_sepconv1 (SeparableConv (None, 16, 16, 728) 536536 block12_sepconv1_act[0][0]

__________________________________________________________________________________________________

block12_sepconv1_bn (BatchNorma (None, 16, 16, 728) 2912 block12_sepconv1[0][0]

__________________________________________________________________________________________________

block12_sepconv2_act (Activatio (None, 16, 16, 728) 0 block12_sepconv1_bn[0][0]

__________________________________________________________________________________________________

block12_sepconv2 (SeparableConv (None, 16, 16, 728) 536536 block12_sepconv2_act[0][0]

__________________________________________________________________________________________________

block12_sepconv2_bn (BatchNorma (None, 16, 16, 728) 2912 block12_sepconv2[0][0]

__________________________________________________________________________________________________

block12_sepconv3_act (Activatio (None, 16, 16, 728) 0 block12_sepconv2_bn[0][0]

__________________________________________________________________________________________________

block12_sepconv3 (SeparableConv (None, 16, 16, 728) 536536 block12_sepconv3_act[0][0]

__________________________________________________________________________________________________

block12_sepconv3_bn (BatchNorma (None, 16, 16, 728) 2912 block12_sepconv3[0][0]

__________________________________________________________________________________________________

add_10 (Add) (None, 16, 16, 728) 0 block12_sepconv3_bn[0][0]

add_9[0][0]

__________________________________________________________________________________________________

block13_sepconv1_act (Activatio (None, 16, 16, 728) 0 add_10[0][0]

__________________________________________________________________________________________________

block13_sepconv1 (SeparableConv (None, 16, 16, 728) 536536 block13_sepconv1_act[0][0]

__________________________________________________________________________________________________

block13_sepconv1_bn (BatchNorma (None, 16, 16, 728) 2912 block13_sepconv1[0][0]

__________________________________________________________________________________________________

block13_sepconv2_act (Activatio (None, 16, 16, 728) 0 block13_sepconv1_bn[0][0]

__________________________________________________________________________________________________

block13_sepconv2 (SeparableConv (None, 16, 16, 1024) 752024 block13_sepconv2_act[0][0]

__________________________________________________________________________________________________

block13_sepconv2_bn (BatchNorma (None, 16, 16, 1024) 4096 block13_sepconv2[0][0]

__________________________________________________________________________________________________

conv2d_3 (Conv2D) (None, 8, 8, 1024) 745472 add_10[0][0]

__________________________________________________________________________________________________

block13_pool (MaxPooling2D) (None, 8, 8, 1024) 0 block13_sepconv2_bn[0][0]

__________________________________________________________________________________________________

batch_normalization_3 (BatchNor (None, 8, 8, 1024) 4096 conv2d_3[0][0]

__________________________________________________________________________________________________

add_11 (Add) (None, 8, 8, 1024) 0 block13_pool[0][0]

batch_normalization_3[0][0]

__________________________________________________________________________________________________

block14_sepconv1 (SeparableConv (None, 8, 8, 1536) 1582080 add_11[0][0]

__________________________________________________________________________________________________

block14_sepconv1_bn (BatchNorma (None, 8, 8, 1536) 6144 block14_sepconv1[0][0]

__________________________________________________________________________________________________

block14_sepconv1_act (Activatio (None, 8, 8, 1536) 0 block14_sepconv1_bn[0][0]

__________________________________________________________________________________________________

block14_sepconv2 (SeparableConv (None, 8, 8, 2048) 3159552 block14_sepconv1_act[0][0]

__________________________________________________________________________________________________

block14_sepconv2_bn (BatchNorma (None, 8, 8, 2048) 8192 block14_sepconv2[0][0]

__________________________________________________________________________________________________

block14_sepconv2_act (Activatio (None, 8, 8, 2048) 0 block14_sepconv2_bn[0][0]

__________________________________________________________________________________________________

global_average_pooling2d (Globa (None, 2048) 0 block14_sepconv2_act[0][0]

__________________________________________________________________________________________________

dense (Dense) (None, 1024) 2098176 global_average_pooling2d[0][0]

__________________________________________________________________________________________________

dense_1 (Dense) (None, 2) 2050 dense[0][0]

==================================================================================================
