# Supplementary material for: Automated system for diagnosing endometrial cancer by adopting deep-learning technology in hysteroscopy
Source: PLoS One. 2021 Mar 31;16(3):e0248526. doi: 10.1371/journal.pone.0248526 (PMC8011803; doi:10.1371/journal.pone.0248526)
Supplement: S8 Table — (DOCX) [file pone.0248526.s009.docx]

**TableS8 : Average accuracies obtained through image-by-image-based predictions grouped in terms of dataset and network types**

|  | Efficient  Net B0 | Mobile  Net V2 | Xception | Ave | MaxAve | MinAve | Gap |
| --- | --- | --- | --- | --- | --- | --- | --- |
| Set X | 0.7895 | 0.7912 | 0.7869 | 0.7892 | 0.8093 | 0.7892 | 0.0201 |
| Set Y | 0.8091 | 0.8121 | 0.8069 | 0.8093 |  |  |  |
| Ave | 0.7993 | 0.8016 | 0.7969 |  |  |  |  |
| MaxAve | 0.8016 | | |  |  |  |  |
| MinAve | 0.7969 | | |  |  |  |  |
| Gap | 0.0047 | | |  |  |  |  |
