# Supplementary material for: Automated system for diagnosing endometrial cancer by adopting deep-learning technology in hysteroscopy
Source: PLoS One. 2021 Mar 31;16(3):e0248526. doi: 10.1371/journal.pone.0248526 (PMC8011803; doi:10.1371/journal.pone.0248526)
Supplement: S9 Table — (DOCX) [file pone.0248526.s010.docx]

**TableS9 : Average accuracies obtained through video-unit-based predictions grouped in terms of dataset and network types**

|  | Efficient  Net B0 | Mobile  Net V2 | Xception | Ave | MaxAve | MinAve | Gap |
| --- | --- | --- | --- | --- | --- | --- | --- |
| Set X | 0.8493 | 0.8373 | 0.8318 | 0.8395 | 0.8914 | 0.8395 | 0.0519 |
| Set Y | 0.8839 | 0.8871 | 0.9031 | 0.8914 |  |  |  |
| Ave | 0.8666 | 0.8622 | 0.8675 |  |  |  |  |
| MaxAve | 0.8675 | | |  |  |  |  |
| MinAve | 0.8622 | | |  |  |  |  |
| Gap | 0.0052 | | |  |  |  |  |
