# Supplementary material for: Automated system for diagnosing endometrial cancer by adopting deep-learning technology in hysteroscopy
Source: PLoS One. 2021 Mar 31;16(3):e0248526. doi: 10.1371/journal.pone.0248526 (PMC8011803; doi:10.1371/journal.pone.0248526)
Supplement: Attachment — Submitted filename: Response to Reviewers .docx [file pone.0248526.s011.docx]

**Response to Editors and Reviewers**

**Journal Requirements:**

When submitting your revision, we need you to address these additional requirements.

**Comment1**

Please ensure that your manuscript meets PLOS ONE's style requirements, including those for file naming. The PLOS ONE style templates can be found at

[https://journals.plos.org/plosone/s/file?id=wjVg/PLOSOne_formatting_sample_main_body.pdf](about:blank) and

[https://journals.plos.org/plosone/s/file?id=ba62/PLOSOne_formatting_sample_title_authors_affiliations.pdf](about:blank)

**Response 1**

**We appreciate your critical comments and useful suggestions. We have reviewed** **PLOS ONE's style requirements and revised the manuscript accordingly.**

**Comment2**

Thank you for stating the following in the Competing Interests section:

"Kenbun Sone has a joint research agreement with Predicthy LLC. The other authors have no competing interests to disclose"

We note that one or more of the authors are employed by a commercial company: Predicthy LLC.

2.1. Please provide an amended Funding Statement declaring this commercial affiliation, as well as a statement regarding the Role of Funders in your study. If the funding organization did not play a role in the study design, data collection and analysis, decision to publish, or preparation of the manuscript and only provided financial support in the form of authors' salaries and/or research materials, please review your statements relating to the author contributions, and ensure you have specifically and accurately indicated the role(s) that these authors had in your study. You can update author roles in the Author Contributions section of the online submission form.

Please also include the following statement within your amended Funding Statement.

“The funder provided support in the form of salaries for authors [insert relevant initials], but did not have any additional role in the study design, data collection and analysis, decision to publish, or preparation of the manuscript. The specific roles of these authors are articulated in the ‘author contributions’ section.”

If your commercial affiliation did play a role in your study, please state and explain this role within your updated Funding Statement.

2.2. Please also provide an updated Competing Interests Statement declaring this commercial affiliation along with any other relevant declarations relating to employment, consultancy, patents, products in development, or marketed products, etc.

Within your Competing Interests Statement, please confirm that this commercial affiliation does not alter your adherence to all PLOS ONE policies on sharing data and materials by including the following statement: "This does not alter our adherence to  PLOS ONE policies on sharing data and materials.” (as detailed online in our guide for authors http://journals.plos.org/plosone/s/competing-interests) . If this adherence statement is not accurate and  there are restrictions on sharing of data and/or materials, please state these. Please note that we cannot proceed with consideration of your article until this information has been declared.

Please include both an updated Funding Statement and Competing Interests Statement in your cover letter. We will change the online submission form on your behalf.

Please know it is PLOS ONE policy for corresponding authors to declare, on behalf of all authors, all potential competing interests for the purposes of transparency. PLOS defines a competing interest as anything that interferes with, or could reasonably be perceived as interfering with, the full and objective presentation, peer review, editorial decision-making, or publication of research or non-research articles submitted to one of the journals. Competing interests can be financial or non-financial, professional, or personal. Competing interests can arise in relationship to an organization or another person. Please follow this link to our website for more details on competing interests: [http://journals.plos.org/plosone/s/competing-interests](about:blank)

**Response 2**

**We appreciate your critical comments and useful suggestions.** **Two co-authors are members of Predicthy LLC and have a joint research contract with us; however, there is no exchange of money such as research funds. Consequently,** **the other authors have no competing interests to disclose.**

3. PLOS requires an ORCID iD for the corresponding author in Editorial Manager on papers submitted after December 6th, 2016. Please ensure that you have an ORCID iD and that it is validated in Editorial Manager. To do this, go to ‘Update my Information’ (in the upper left-hand corner of the main menu), and click on the Fetch/Validate link next to the ORCID field. This will take you to the ORCID site and allow you to create a new iD or authenticate a pre-existing iD in Editorial Manager. Please see the following video for instructions on linking an ORCID iD to your Editorial Manager account: [https://www.youtube.com/watch?v=_xcclfuvtxQ](about:blank)

**Response 3**

**We appreciate your critical comments and useful suggestions.**

**My ORCID iD is as follows: https://orcid.org/0000-0002-7218-6401.**

**I have validated it in Editorial Manager, as per your instructions.**

**Reviewer #1:**

This work aims to establish deep learning models for classifying the presence of endometrial tumors in hysteroscopic images. And an average diagnostic accuracy exceeding 90% was realized when using the combination of 72 trained DNN models. However, I have the following concerns:

**Comment1**

I am a bit curious why they use this deep learning architecture for endometrial tumors detection, rather than shallow machine learning models.

**Response 1**

**We appreciate your critical comments and useful suggestions. Deep learning is highly anticipated in the medical field because deep learning techniques are particularly suitable for image analysis. They can be used for classification, image quality improvement, and segmentation of medical images.** **In contrast, shallow machine learning is not suitable for image recognition. We have added this information to the revised manuscript considering your comment (Lines 66-69).**

**Comment2**

There are several errors in this manuscript, such as “The corresponding sensitivity and specificity equaled 91.66% and 89.36, respectively”. Is it 89.36%? The authors should double check the manuscript.

**Response 2**

**We appreciate your critical comments and useful suggestions. It is 89.36%（Lines36）. We have corrected the oversight.**

**Comment3**

The manuscript should give the overall model architecture.

**Response 3**

**We appreciate your critical comments and useful suggestions. We have added the overall architecture of the model (Figure2) in accordance with your suggestion.**

**Comment4**

The metric method for the model is too simple, the author should add more metric method. Please refer to several literatures, such as:

Pang Shanchen, Ding Tong, Qiao Sibo, Meng Fan, Wang Shuo, Li pibao, WangXun . A novel YOLOv3-arch model for identifying cholelithiasis and classifying gallstones on CT images,2019, Plos one, 6(14):e0217647.DOI: 10.1371

Wang Shudong, Dong Liyuan, Wang Xun, Wang Xingguang. Classification of Pathological Types of Lung Cancer from CT Images by Deep Residual Neural Networks with Transfer Learning Strategy. Open Medicine, 2020, 15(1): 190-197.

Shanchen Pang, Yaqin Zhang, Mao Ding, Xun Wang, Xianjin Xie. A Deep Model for Lung Cancer Type Identification by Densely Connected Convolutional Networks and Adaptive Boosting. IEEE Access 2020,8: 4799-4805.

Shanchen Pang, Fan Meng, Xun Wang, et al. VGG16-T: A Novel Deep Convolutional Neural Network with Boosting to Identify Pathological Type of Lung Cancer in Early Stage by CT Images, International Journal of Computational Intelligence Systems. Vol.13(1), pp. 771-780, 2020.

**Response 4**

**We appreciate your critical comments and useful suggestions. We have added the metric methods in accordance with your comments. F-score and Precision have been added to Table 2. In addition, the description and structure of each network are also given (Tables S4, S5, S6, S7, Lines 164-179).**

**Reviewer #2:**

In the paper, authors present an artificial-intelligence-based system to detect the regions affected by endometrial cancer automatically from hysteroscopic images. The diagnosis accuracy is increased. However, there are some details that can be improved.

**Comment1**

The models used in the paper are not presented well.

**Response 1**

**We appreciate your critical comments and useful suggestions. We have added the overall architecture of the model (Fgure2) to provide further details of the model used. In addition, the description and structure of each network are also given (Tables S4, S5, S6, S7, Lines 164-179).**

**Comment2**

The set of threshold value is 50, maybe you can explain some details about that.

**Response 2**

**We appreciate your critical comments and useful suggestions. The threshold was taken from the points where the malignant score intersects with the other scores rather than the point where the average of two scores was the best, because the threshold should be set lower to reduce oversight cases in the actual clinical devices. We have added this information to the revised manuscript considering your comment (Lines 152-154).**

**Comment3**

The writing of the paper should be taken care. For example, the text size on the tables, the text-transform on subtitle of page 7.

**Response 3**

**We appreciate your critical comments and useful suggestions. We have revised the manuscript in accordance with your suggestion and PLOS ONE's style requirements.**
